# Acute Intake of a Grape and Blueberry Polyphenol-Rich Extract Ameliorates Cognitive Performance in Healthy Young Adults During a Sustained Cognitive Effort

**DOI:** 10.3390/antiox8120650

**Published:** 2019-12-17

**Authors:** Pierre Philip, Patricia Sagaspe, Jacques Taillard, Claire Mandon, Joël Constans, Line Pourtau, Camille Pouchieu, Donato Angelino, Pedro Mena, Daniela Martini, Daniele Del Rio, David Vauzour

**Affiliations:** 1 Pôle Neurosciences Cliniques, Centre Hospitalier Universitaire de Bordeaux, F-33076 Bordeaux, France; pierre.philip@chu-bordeaux.fr (P.P.); patricia.sagaspe@chu-bordeaux.fr (P.S.); 2 Sommeil, Addiction et NeuroPSYchiatrie, Université de Bordeaux, CNRS, SANPSY, USR 3413, F-33000 Bordeaux, France; jacques.taillard@u-bordeaux.fr; 3 Centre d’Investigation Clinique Bordeaux, INSERM CIC 1401, Centre Hospitalier Universitaire de Bordeaux, F-33000 Bordeaux, France; 4 Vascular Medicine Service, Centre Hospitalier Universitaire de Bordeaux, F-33000 Bordeaux, France; claire.mandon@chu-bordeaux.fr (C.M.); joel.constans@chu-bordeaux.fr (J.C.); 5 Activ’Inside, F-33750 Beychac et Caillau, France; l.pourtau@activinside.com (L.P.); c.pouchieu@activinside.com (C.P.); 6 Department of Food & Drugs, University of Parma, 43125 Parma, Italy; donato.angelino@unipr.it (D.A.); pedromiguel.menaparreno@unipr.it (P.M.); 7 Department of Veterinary Science, University of Parma, 43125 Parma, Italy; daniela.martini@unipr.it (D.M.); daniele.delrio@unipr.it (D.D.R.); 8 School of Advanced Studies on Food and Nutrition, University of Parma, 43125 Parma, Italy; 9 Microbiome Research Hub, University of Parma, 43124 Parma, Italy; 10 Norwich Medical School, Biomedical Research Centre, Faculty of Medicine and Health Sciences, University of East Anglia, Norwich NR4 7TJ, UK

**Keywords:** single dose, flavonoids, flavanols, monomers, brain, epicatechin, catechin, cognition, human

## Abstract

Despite an increasing level of evidence supporting the individual beneficial effect of polyphenols on cognitive performance, information related to the potential synergistic action of these phytonutrients on cognitive performance during a prolonged cognitive effort is currently lacking. This study investigated the acute and sustained action of a polyphenols-rich extract from grape and blueberry (PEGB), on working memory and attention in healthy students during a prolonged and intensive cognitive effort. In this randomised, cross-over, double blind study, 30 healthy students consumed 600 mg of PEGB or a placebo. Ninety minutes after product intake, cognitive functions were assessed for one hour using a cognitive demand battery including serial subtraction tasks, a rapid visual information processing (RVIP) task and a visual analogical scale. Flow-mediated dilation (FMD) and plasma flavan-3-ols metabolites quantification were also performed. A 2.5-fold increase in serial three subtraction variation net scores was observed following PEGB consumption versus placebo (*p* < 0.001). A trend towards significance was also observed with RVIP percentage of correct answers (*p* = 0.058). No treatment effect was observed on FMD. Our findings suggest that consumption of PEGB coupled with a healthy lifestyle may be a safe alternative to acutely improve working memory and attention during a sustained cognitive effort.

## 1. Introduction

With a multitude of exams, evaluations, and deadlines, university students are experiencing enormous pressure to perform. These stressful situations have a critical impact on learning and memory processes [1], and therefore students are seeking solutions to ameliorate their cognitive capabilities over short period of time and particularly during exams. In this context, and in order to improve their academic performance or productivity, students often self-report the misuse of pharmaceutical stimulants, such as amphetamines or dextroamphetamines, originally prescribed for attention-deficit/hyperactivity disorder symptoms [2,3]. These uncontrolled medicines and practices involve enormous public safety issues possibly leading to major health complications. Hence, it is crucial to propose quick and safe alternative solutions for cognitive performance enhancement and maintenance during a cognitively demanding effort.

Lifestyle strategies such as nutritional interventions have received increased attention as they provide safe and effective solutions to improve cognitive performance [4]. In particular, dietary polyphenols, plant-derived compounds found abundantly in fruits, vegetables, cocoa, and certain beverages such as tea [5], by involving cellular and molecular mechanisms are able to enhance cognitive functions following acute and chronic interventions in both humans [6,7,8] and animals [9,10,11]. For example, a higher intake of polyphenol-rich foods and beverages was associated with better cognitive functions, including semantic and episodic memory in older adults [12,13,14,15] and middle-aged population [16,17]. Furthermore, dietary intervention trials have shown that daily supplementation with blueberry or grape-derived products, particularly rich in anthocyanins and flavan-3-ols, can improve learning and memory in both healthy or mild cognitive impairment diagnosed older adults [18,19]. We previously showed that a polyphenol-rich extract made from grape and blueberry (PEGB) was able to attenuate cognitive decline and to improve neuronal function in aged mice [20]. Furthermore, we also reported improved working memory in a subgroup of participants with advanced cognitive decline following six months supplementation with a PEGB, although such results were not replicated in healthier older adults. Importantly, these cognitive improvements were positively correlated with urinary concentration of native and conjugated metabolites mainly derived from the flavan-3-ols monomers, catechin, and epicatechin [15,21].

Flavan-3-ols and their plasma metabolites have been reported to reach their maximal concentration between 1 and 2 h (median 1.4 h) post consumption [22], with such timing keeping in line with observed improvements in cognitive performance [6]. Despite an increasing level of evidence supporting the beneficial acute impact of polyphenols on global cognitive performance [23,24,25], only a few studies have investigated the sustained action of these dietary bioactives during a prolonged cognitive effort. Notably, Scholey and colleagues reported that cocoa flavan-3-ols were able to improve executive function in young adults when employing a sustained mental effort battery designed to reduce ceiling effects [25]. Similarly, Whyte et al. concluded that anthocyanin rich freeze-dried wild blueberries could enhance executive function during demanding elements of a cognitive task in 7 to 10 year-old children [26]. However, the synergistic effect of a grape and blueberry polyphenols rich-extract on sustained cognitive performance during a prolonged cognitive effort has never been investigated.

Measures of cerebral and peripheral vascular functions have previously been correlated with cognitive function [27] and recent studies have reported improvements in flow-mediated dilation (FMD) measures following flavanols intake [28,29]. Therefore, the present study aimed at evaluating healthy students working memory and attention performances over the course of a sustained cognitive challenge occurring ninety minutes after acute administration of PEGB. Underlying mechanisms of action supporting the effect were investigated through FMD measures and the quantification of circulating phase-2 metabolites deriving from the flavan-3-ols monomers.

## 2. Materials and Methods

### 2.1. Ethical Approval and Consent to Participate

The study was conducted in accordance with the Declaration of Helsinki and the French Public Health Code. The study protocol was approved by an ethical review committee for people’s protection (CPP Sud Méditerranée IV, 10/10/2017; ID-RCB: 2017-A02112-51; ClinicalTrial.gov Identifier: NCT03508206). All subjects provided their written informed consent for inclusion before they participated in the study.

### 2.2. Study Participants

**Figure 1:**
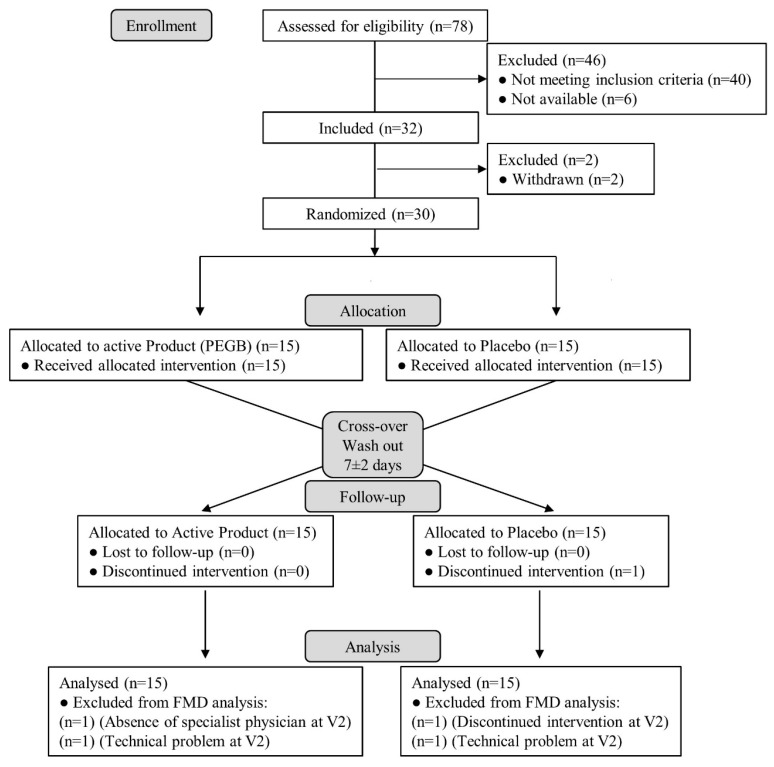
Consolidated Standards of Reporting Trials (CONSORT) flowchart diagram. FMD: flow-mediated dilation; V1: visit 1; V2: visit 2.

Thirty healthy male and female students, aged between 18 and 25 years with exams at least every 6 months and attending lectures equivalent to at least 2 full days per week, were recruited between November 2017 and May 2018, from the University of Bordeaux (Bordeaux, France) and enrolled by the SANPSY Unit (Bordeaux) in this study (See Figure 1 for the Consolidated Standards of Reporting Trials (CONSORT) diagram). Individuals with high blood pressure (BP > 140/90 mmHg), body mass index (BMI) >30 kg/m^2^, active smokers, history or currently suffering from psychiatric or neurologic disorders, diabetes mellitus, dyslipidaemia, cardiovascular disease, thyroid disorders, or taking antidepressant, neuroleptic, hypnotic, anxiolytic, anti-hypertensive, anticoagulant or veinotonic treatments, or subjected to anaesthesia in the last 7 days were excluded from the study. Other exclusion criteria were restrictive or unbalanced diet, excessive alcohol consumption (>15 units/week) or either consuming food supplements aiming at improving memory, concentration, sleep, stress, anxiety or containing ingredients derived from grape, cranberry, bilberry, tea, coffee bean, citrus, pine, olive, omega-3 fatty acids, ginkgo biloba, Asiatic ginseng, multivitamin, and caffeine.

Participants were deemed eligible following a clinical physical examination that included BP and BMI. Subjects refrained from consuming polyphenol-rich foods for 24 h before each testing visit. In particular, the following food and drinks were excluded from the participants’ diets: red fruits, red fruit juices, tea or any herbal infusion, dark chocolate (≥70% cocoa), and energy drink.

### 2.3. Tested Product

Participants were asked to either consume 600 mg of polyphenol-rich active extract made from grape (*Vitis vinifera L*.) and wild blueberry (*Vaccinium angustifolium*) (Memophenol™, Activ’Inside, Beychac et Caillau, France), or a placebo containing pure maltodextrin (Maltrin^®^ M100, Roquette, Lestrem, France) and providing no polyphenol. The 600 mg dose of PEGB was chosen based on our previously published preclinical and clinical studies [11,21] and was given as 2 × 300 mg capsules which contained low-molecular weight polyphenols. Details of the polyphenolic content of our extract are provided in Table 1. Both active and placebo capsules were formulated in a single batch, included in externally similar capsules and without taste differences to avoid unblinding. Capsules were provided on the visit day in opaque pill dispensers.

### 2.4. Study Design

A randomised, double-blind (neither participants nor investigators had access to the nature of the product tested), placebo-controlled cross-over design protocol was performed. Each participant went through one inclusion visit (V0) followed by two testing visits (V1 and V2) during which they either consumed the active product or a placebo. Following eligibility checks for inclusion (V0), participants were randomly assigned to a sequence order of treatments. The randomisation process was based on computer generated codes that were used in a sequential order and established by an independent person, neither participating in the clinical phase, nor in the processing of the study data. The randomisation was equilibrated by block size of 4. Participants were asked not to change their feeding, sleeping, and exercise habits during the whole study duration (3 weeks). Participants performed a training session on the computerised cognitive demand battery (CDB) for habituation and detection of individual problems in task realisation. At the end of the inclusion visit, participants were provided with sleep diaries.

**Figure 2:**
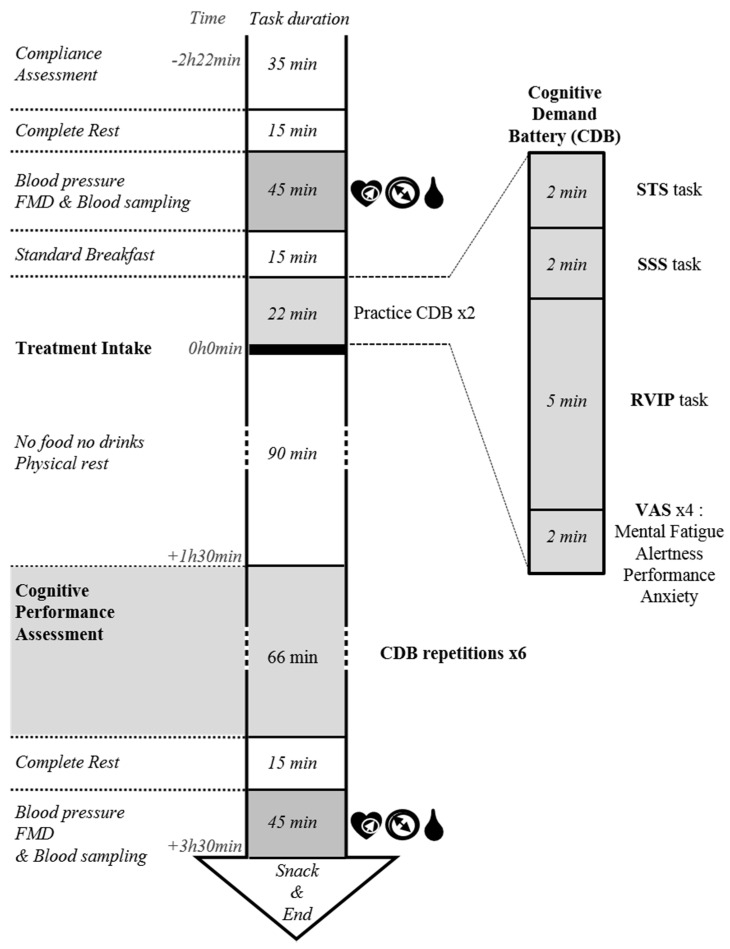
Testing visits schedule. Participant compliance was controlled at the beginning of each testing visit. After evaluation of cardiovascular parameters and blood sampling, they were served with a standardised breakfast and exposed to the cognitive demand battery (CDB) twice for practice before treatment intake. After a 90 min at rest (absorption period), subjects were transferred in a dedicated room for 66 min of intensive cognitive challenge comprising 6 consecutive CDB repetitions in order to test attention and working memory through a serial three subtraction task (STS), a serial seven subtraction task (SSS), a rapid visual information processing task (RVIP), and subjective ratings using visual analogical scales (VAS). After a second set of cardiovascular measures and blood sampling, subject was having a snack before terminating the visit.

The V1 visit was planned 7 ± 2 days after the inclusion visit, while the V2 visit was planned following a wash-out period (7 ± 2 days). Briefly, in the morning of each testing visits, volunteers were asked to arrive at the clinical facility following a 12 h fasting period. After diaries checks, absence of recent consumption of cannabis/alcohol was confirmed by a urine tetrahydrocannabinol test and breath alcohol test. Research coordinators then randomly assigned eligible participants to a treatment sequence order. Subjects were next taken to a clinical exam dedicated room and asked to rest for 15 min in a supine position prior to basal measurements of heart rate (HR), blood pressure (BP), flow mediated dilation (FMD), and blood sampling (BS). Then, they were served with a caffeine-free, low flavonoid and glucose balanced standard breakfast before carrying out a training session on CDB (2 × 11 min). Subsequently, subjects were asked to consume the allocated treatment with a glass of water and to rest for 90 min in a quiet room prior to starting a 66 min intensive and cognitively demanding test session (six consecutive CDB repetitions). At the end of the test session and following a 15 min rest, participants underwent the second HR, BP, FMD, and BS session (Figure 2).

### 2.5. Cognitive Assessment

Participants were cognitively challenged over 66 min with an intensive and cognitively demanding series of tasks requiring different cognitive functions, especially working memory and attention, through the Computerised Mental Performance Assessment System (COMPASS, Northumbria University, Newcastle upon Tyne, UK). The task consisted in 6 repetitions of a 11 min long CDB blocks. Each block was composed of a serial three subtraction task (STS), a serial seven subtraction task (SSS), a rapid visual information processing task (RVIP), and subjective ratings using visual analogical scales (VAS) [30,31]. During the STS task, participants had to count quickly and accurately backwards in threes from a given random starting number between 800 and 999 presented on the screen. Answers were dialled on the computer keypad. The task was scored for number of total answers, number of correct answers and number of errors. From raw scores, a net score (number of correct answers-number of errors) and percentage of correct answers (number of correct answers/number of total answers) were calculated. The duration of this task was 2 min. The SSS task was identical to the STS with the exception that it involved serial subtraction of sevens. These two subtraction tasks aimed at evaluating subjects working memory and attention levels along with the challenge [32].

For the RVIP task, participants were required to monitor a continuous series of digits for targets of three consecutive odd or three consecutives even digits. Digits were presented at the rate of 100 per minute on a computer screen and the volunteer responded to the detection of a target string by pressing the ‘space bar’ as quickly as possible. The task was scored for percentage of target strings correctly detected, average reaction time for correct detections, and number of false alarms. The task lasted for 5 min and aimed at measuring subjects sustained attention and working memory along with CDB repetitions.

For the VAS, participants rated their current subjective mental fatigue, alertness, anxiety and their cognitive performance state by making a mark on four individual 100 mm visual analogue scale with the end points labelled “not at all” (left hand end) and “very much so” (right hand end). A maximum of thirty seconds was allowed to complete each VAS scoring.

### 2.6. Biological Samples Analysis

For identification and quantification of plasma flavan-3-ols and their gut-derived metabolites, blood samples were drawn twice per visit in EDTA tubes. The first sample was obtained just upon arrival (around 08:30) and following a 12h fast, whilst the second sample was drawn after the second FMD assessment (3.5 h after treatment post-absorption). Samples were immediately processed for plasma by 5 min centrifugation at 1600× *g*, at 4 °C, aliquoted and stored at −80 °C until analysis. Phenolic metabolites were then extracted and concentrated using a solid phase extraction (SPE) method previously reported [33] and were analysed by a UHPLC DIONEX Ultimate 3000 equipped with a triple quadrupole TSQ mass spectrometer (Thermo Fisher Scientific Inc., San José, CA, USA) fitted with a heated-ESI (H-ESI) (Thermo Fisher Scientific Inc., San José, CA, USA) probe. Chromatographic separation, ionisation parameters, and spectrometric characteristics of the considered compounds were set as previously reported [34,35]. Quantification was performed with calibration curves of standards, when available or using the most structurally similar compound [34,35]. Data processing was performed using Xcalibur software (Thermo Scientific Inc., Waltham, MA, USA).

### 2.7. Heart Rate, Blood Pressure, and Flow-Mediated Dilation Assessments

These measurements were taken twice per visit at the exact same time, location, and temperature conditions by a treatment blinded examiner. Upon arrival participants were left to rest for 15 min in a supine position. HR, diastolic (DBP) and systolic (SBP) blood pressures (safety parameters) were determined by calculating the mean of 3 consecutive measures. Then subject right arm was immobilised in an appropriate support and the brachial artery diameter was recorded by fixing a 10MHz linear probe (ultrasound transducer Vinno™ E10; probe F4-12, Vinno France, Palaiseau, France) above the segment of interest. In order to limit intra and inter-visit measures variability, probe position was recorded by measuring the distance between the antecubital fossa and probe distal border. Ischemia was induced with an inflatable air cuff placed around the forearm just above the wrist. Baseline artery diameter was measured for 2 min (4 × 30 s), followed by the air cuff-induced ischemia (250 mm Hg for 5 min). Immediately after pressure release, hyperaemia-induced changes in arterial diameter were recorded for 2 min (4 × 30 s). Finally, ultrasound transducer signal was processed and analysed using semi-automated border detection (Brachial Analyser 5; Medical Imaging Application LLC, Coralville, IA, USA). Post-ischemia FMD (4 × 30 s segments) was expressed as the percentage of basal pre-ischemia diameter (2 min mean). The post-ischemia segment with the higher percentage of dilation was considered as the FMD peak, theoretically located between 30 and 60 s [36].

### 2.8. Statistics

Sample size calculations were based on the results obtained by Scholey et al. on the acute effect of flavonoids on cognitive performances in healthy students [25]. Using the G*Power 3 software [37] (University of Düsseldorf, Düsseldorf, Germany) and employing an a priori approach, our analysis indicated that in order to detect a significant difference between PEGB and the placebo of 5.5 (SD 7.5) on working memory, 30 participants would be necessary to achieve a two-sided 0.05 significance level at a power of 0.80.

One subject discontinued the study due to family health issues leading to missing data completely at random at visit 2 for all cognitive tests, heart rate, FMD, and blood pressures. Missing cognitive data were replaced by the mean of the observed values to limit the lack of statistical power. Normal distribution of outcomes was evaluated via Shapiro–Wilk normality plot tests. A preliminary analysis on the first CDB score was performed with a linear mixed model with treatment (placebo, PEGB), treatment allocation order (1: placebo/PEGB, 2: PEGB/placebo), and treatment x order as fixed effects. No significant order × treatment interaction was found but a significant order effect was observed for the number of correct STS responses and RVIP false alarms. In order to evaluate variations in cognitive performance for the entire duration of the cognitive challenge, raw scores from STS, SSS, RVIP, and VAS along the different CDB repetitions are expressed as the change from the first score. Linear mixed models were then used to model the effect of treatment, repetition and (treatment × repetition) in all cognitive tests with treatment (placebo, PEGB), treatment allocation order (1, 2), repetition (22, 33, 44, 55, and 66 min) and treatment × repetition as fixed effects. Multiple pairwise comparisons with Bonferroni correction were applied when appropriate. A potential gender effect on cognitive tests has been checked but no effect was found. The effect of treatment on heart rate, blood pressure, and FMD measures were also analysed with linear-mixed models. For the FMD experiments, due to missing data (recording interruption, technical issues), 4 participants were excluded from the analyses, leading to a total number of 26 participants for this aspect of the study. Flavan-3-ols metabolites post-treatment were analysed using paired t-tests. All statistical tests were 2-sided, and a *p*-value < 0.05 was considered statistically significant. All data were analysed using SAS version 9.3 (SAS Institute, Cary, NC, USA). Figures were computed with GraphPad Prims (version 6.01, GraphPad Software, San Diego, CA, USA).

## 3. Results

### 3.1. Inclusion and Population Characteristics

The study population consisted of 30 healthy male (*n* = 14) and female (*n* = 16) students with a mean age of 22 ± 1.7 years. All participants reported being in a good health, not consuming any food supplements, medications, or illicit drugs that would interfere with the tested product. All baseline values were within the physiological range (Table 2). The test capsules were well tolerated, and no adverse effects were reported during the study. Except for one participant, who had family problems, all subjects completed the trial.

### 3.2. PEGB Improves Working Memory Performance

**Figure 3:**
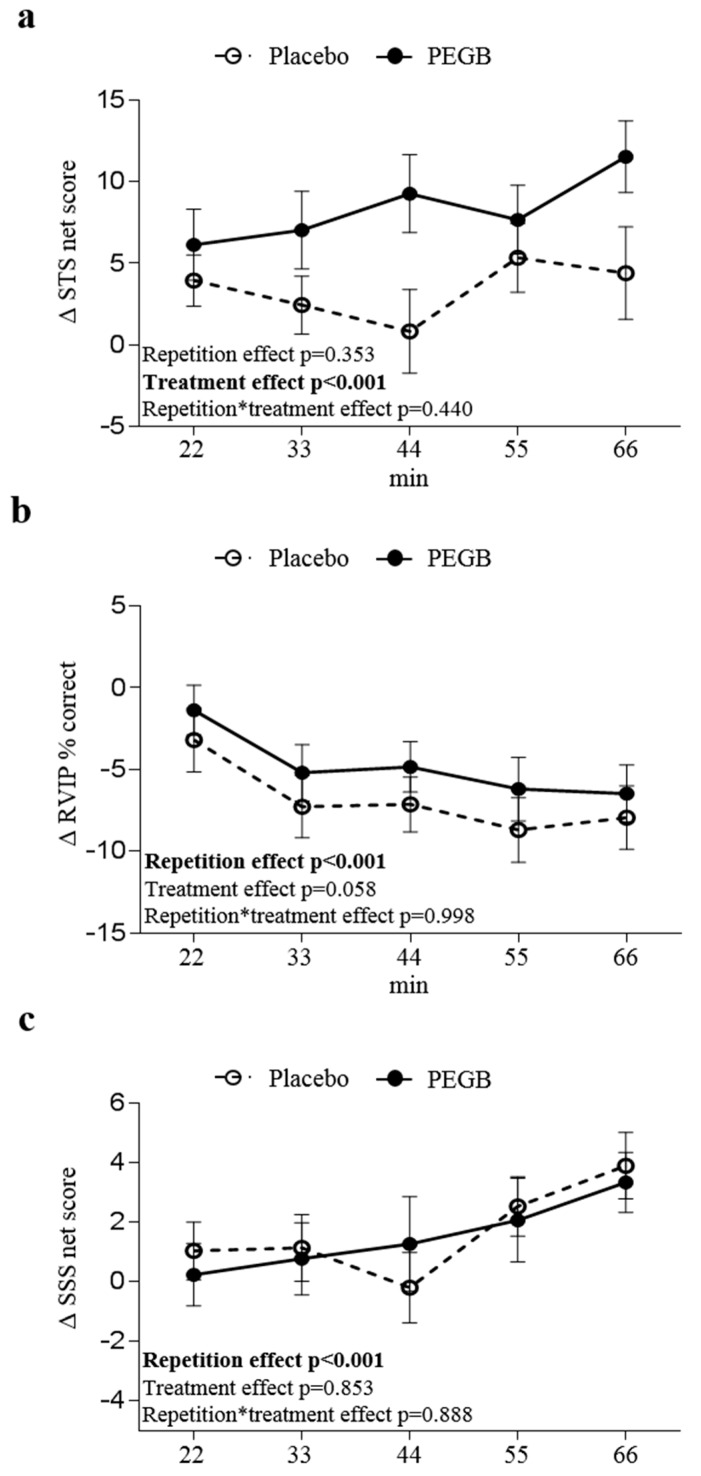
Cognitive net scores (difference from first score, Δ) along the CDB repetitions: (**a**) STS net score, (**b**) RVIP% correct and (**c**) SSS net score. STS and SSS net score were obtained by making the difference between the number of correct answers and the number of errors. A total of six CDB measures (every 11 min) were performed during the cognitive challenge. Data are expressed in mean ± SE. *p*-values generated by the linear-mixed models are reported for the effects of treatment, repetition and treatment x repetition. *p*-values in bold are statistically significant. *n* = 30 for placebo and PEGB group.

Analysis of cognitive performance variation (from the 1st score) during the sustained cognitive effort showed a significant effect of treatment on STS task for the different outcomes (number of total answers (*p* = 0.001), number of correct answers (*p* < 0.001), number of errors (*p* = 0.041), and percentage of correct answers (*p* = 0.001)) (Table 3). In agreement with these results, a significant effect of treatment was also observed on STS net scores (*p* < 0.001, Figure 3a). Indeed, a 2.5-fold increase in STS variation net scores in the PEGB group compared to placebo (8.33 ± 1.00 vs. 3.38 ± 0.99 (mean ± SE)) was found.

Regarding the RVIP task, although the diminution in the percentage of correct answers from the first score was slightly lower in the PEGB group compared to the placebo group (−4.75 ± 1.12 vs. −6.76 ± 1.12 (mean ± SE)) only a trend towards significance was observed (*p* = 0.058). No significant effect of treatment was found for RVIP false alarm and reaction time (Table 3).

No significant treatment effect was observed for changes in SSS scores from the first score (number of total answers, correct answers, and errors, percentage of correct answers and net score) (Table 3 and Figure 3c).

In order to evaluate mean cognitive performance along the cognitive challenge, a complementary analysis for STS, RVIP, and SSS score using incremental approach (described in Figure S1 caption) was performed and showed also a significant effect of PEGB on STS net score and RVIP% correct answers (Figure S1).

### 3.3. PEGB Improves Subjective Rating of Cognitive Performance

**Figure 4:**
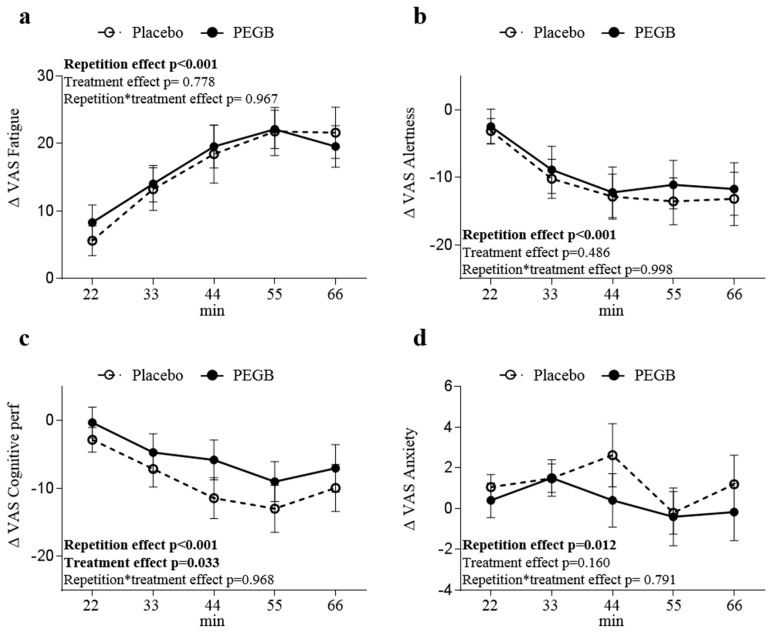
Subjective ratings (difference from first score, Δ) of (**a**) mental fatigue, (**b**) alertness, (**c**) cognitive performance, and (**d**) anxiety using visual analogue scales (VAS) during the CDB repetitions. Data are expressed in mean ± SE. *p*-values generated by the linear-mixed models are reported for the effects of treatment, repetition, and treatment × repetition. *p*-values in bold are statistically significant. *n* = 30 for placebo and PEGB group.

At the end of each CDB repetition, participants were asked to rate their perception of mental fatigue, alertness level, cognitive performance, and anxiety. Independently from treatment, self-perceived mental fatigue was significantly increased along the CDB repetitions, with a marked effect starting from the third repetition (*p* < 0.001) (Figure 4a). Such repetition effect was observed with both self-reported alertness (*p* < 0.001) (Figure 4b) and cognitive performance (*p* < 0.001, Figure 4c). In addition, the self-reported cognitive performance was significantly higher in the PEGB group compared to the placebo group throughout the CDB repetitions (*p* = 0.033, Figure 4c). Finally, there was a significant repetition effect on self-reported anxiety (*p* = 0.012) but neither time × treatment interaction nor treatment effect were observed (Figure 4d).

### 3.4. PEGB Intake Significantly Increase Circulating Flavan-3-ols Metabolites Levels, but Does not Affect Vascular Functions

**Figure 5:**
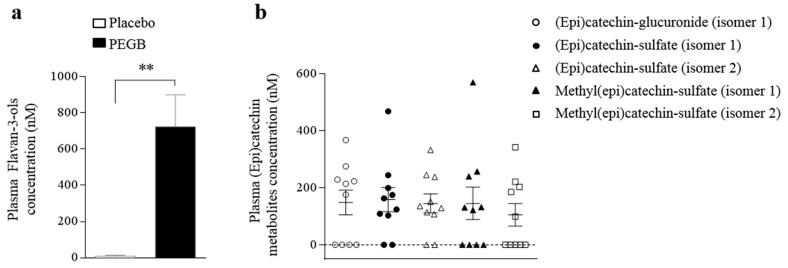
Flavan-3-ols metabolites detected in human plasma samples (*n* = 10): (**a**) plasma concentration of total flavan-3-ols and their colonic metabolites detected 3.5 h after product ingestion; (**b**) individual plasma concentration of (epi)catechin conjugated metabolites detected. Data are expressed as mean ± SE. Significant statistical results for treatment effect from paired *t*-test are shown by (** *p* < 0.01).

PEGB supplementation resulted in a significant increase in the amount of total circulating flavan-3-ols and their gut microbial metabolites 3.5 h following intake (722.7 ± 176.4 nM, *p* < 0.001) (Figure 5a). Among the flavan-3-ols metabolites, 2 (epi)catechin and 2 methyl(epi)catechin sulphate isomers as well as (epi)catechin glucuronide have been identified and quantified, although no significant group differences were observed (Figure 5b). Concerning gut microbial metabolites, 5-(hydroxyphenyl)-γ-valerolactone sulfate isomers (the sum of 5-(3′-hydroxyphenyl)-γ-valerolactone-4′-sulphate and 5-(4′-hydroxyphenyl)-γ-valerolactone-3′-sulphate), 5-phenyl-γ-valerolactone-3ʹ-sulfate, and 4-hydroxy-5-(hydroxyphenyl)valeric acid-sulfate isomers were found in concentrations lower than 100 nM in only a few plasma samples.

**Figure 6:**
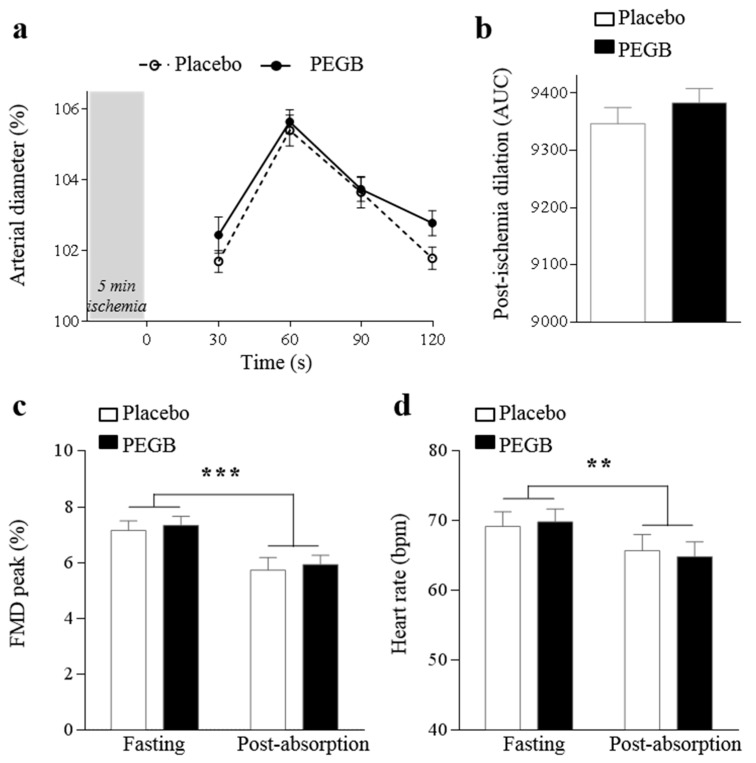
Effect of time and treatment on flow mediated dilation and heart rate during visit: (**a**) percentage of post-ischemia arterial dilation from 30 s to 120 s; (**b**) area under the curve of post-ischemic total dilatation; (**c**) FMD peak; and (**d**) heart rate. Data are expressed as mean ± SE. Significant statistical results for time effect from linear-mixed models are shown by (** *p* < 0.01, *** *p* < 0.001). *n* = 26 for placebo and PEGB group.

No significant effect on the vascular parameters was observed as measured by FMD (Figure 6a,b) and blood pressure monitoring. However, a significant time effect was detected when comparing fasting and post-absorption FMD peak (*p* < 0.001) with a decrease of FMD peak 3.5 h post ingestion (Figure 6c). A similar reduction was observed when comparing heart rate along the visits (*p* = 0.004) (Figure 6d).

## 4. Discussion

In the present study, we investigated the acute effect of a polyphenol rich extract made from grape and blueberries (PEGB) on working memory and attention during a prolonged cognitive challenge in healthy students. Our results demonstrate for the first time that over the course of the cognitive challenge, both working memory and attention were globally improved following PEGB consumption.

Placed in cognitively demanding conditions, participants felt an alteration in mental fatigue, alertness, cognitive performance, and anxiety. Our results are aligned with previous studies employing such paradigms and demonstrate the validity of our protocol [25,38]. Unlike previously reported for cocoa polyphenols, subjective ratings for mental fatigue under PEGB did not reach significance. However, our student cohort reported a positive effect on cognitive performance. Such discrepancy might be related to the product sourcing and composition along with the food bioactives available in our product (flavan-3-ols, flavonols, acid phenolic, stilbene, and anthocyanins versus flavan-3-ols alone).

In addition to visual analogue scales which provide a subjective state measure, we also evaluated the product impact on objective cognitive outcomes using computerised serial subtractions (SSS and STS) and RVIP tasks. A main significant and positive effect of PEGB was observed on STS scores and not on SSS performances. In agreement with our findings, improvements in STS scores and no effect on SSS scores have been previously reported following an acute consumption of 552 or 994 mg cocoa flavanol-3-ols [25]. Although both STS and SSS tests require subtraction-related cognitive processes, the SSS task is more complex due to the difference in subtraction process. Indeed, subtraction of 7′s requires more regrouping (borrowing) than subtraction in 3′s and therefore requires more cognitive demand and different brain region. Moreover, although both tasks have attentional components, SSS tests more heavily rely on working memory and executive function [32]. Further to the STS task, a trend towards an increase in RVIP accuracy without modification of reaction time was observed following PEGB supplementation. Such observations are in agreement with a previous study reporting a better RVIP accuracy after supplementation with a blackcurrant extract [39]. However, such results were not replicated following consumption of cocoa tablets [38]. It is therefore likely that changes in RVIP scores may be related partially to the anthocyanins content of our product, further demonstrating the combined and synergistic effect of the different bioactives present in PEGB.

In order to get insight into the potential mechanisms underlying the cognitive effects of the product, we measured flavan-3-ols metabolites in the systemic circulation. Analysis of ten participants plasma following 600 mg PEGB consumption, revealed the circulating flavan-3-ols metabolites (epi)catechin-glucuronide, (epi)catechin-sulfate, and methyl(epi)catechin-sulfate isomers 3.5 h post ingestion. The observed concentrations are in line with previous studies reporting (epi)catechin and methyl-(epi)catechin metabolites 4 h after flavan-3-ols consumption with a range of 50–250 nM [33,40,41,42] Concerning the flavan-3-ols gut microbial derivatives, low amounts of phenyl-γ-valerolactones and a phenylvaleric acid were found only in a few plasma samples, supporting the large differences in flavan-3-ols metabolism due to inter-individual gut microbiota variability [43]. Results are in agreement with previous data reporting a 2-fold variation in the peak plasma concentration (Cmax values) of structurally related epicatechin metabolites [42]. (Epi)catechin are mainly removed from the circulatory system via urinary excretion, with plasma half time ranging from 1.1 to 2.2 h, while phenyl-γ-valerolactones and phenylvaleric acid Tmax were found to be around 6 h, which may justify the low concentrations found for these microbial metabolites at the 3.5 h collection-time point [40]. In general, the low concentrations of flavan-3-ols metabolites in some participants could be explained by inter-individual differences in their production or faster urinary excretion, although further explorations would be necessary to confirm this hypothesis.

An indirect action through cerebrovascular endothelial function optimisation, leading to cerebral blood flow (CBF) increase may explain cognitive performance improvement [6]. This increase in local perfusion would provide optimal distribution of the essential nutrients for neuronal activity, such as oxygen and glucose [44,45]. In addition, clinical investigations have shown that dietary flavanols intake was associated with improvement in endothelial functions [46,47]. Given that the global cognitive performances were higher and maintained up to 2.5 h following the active product ingestion, we queried whether such improvements may be the results of vascular changes. In order to address this question, we assessed blood flow changes via FMD measures on the brachial artery. Although previous studies have reported increased FMD values 2 h following flavanol-rich products consumption [6], no difference on FMD measures were observed between our treatment groups. Such discrepancy may be explained by the fact that FMD values have diurnal variations and are sensitive to mental fatigue [48]. Indeed, exposure to an acute mental stress can reduce FMD by 2.8%, an effect sustained up to 4 h [49]. Therefore, one could imagine that exposure to CDB repetitions during our “exam-like” situation could have induced a sufficient mental stress to attenuate FMD levels, therefore masking the possible effect of PEGB.

Although not investigated in this study, other potential mechanisms to improve cognitive functions may include a direct action of (epi)catechin metabolites on neurons through early LTP mechanisms [11,50]. Indeed, previous rodents studies have demonstrated that (epi)catechin metabolites can cross the blood brain barrier following chronic grape flavan-3-ols monomer extract supplementation [51] or after acute ^14^C-(epi)catechin feeding [52]. It has been shown that the levels of monoamines, a crucial family of neurotransmitter increased during working memory and attention tasks [53], can be modulated by blackcurrant supplementation through monoamine oxidase enzyme inhibition leading to improved attention [39]. Finally, emerging evidence supports a beneficial action of similar concentration of flavan-3-ols on insulin sensitivity suggesting that blood glucose regulation may provide an additional effect on cognitive function [54]. However, in this study, because the breakfast was not consumed in conjunction with the polyphenol-rich extract, it was impossible to conclude on the glucose regulation as a surrogate marker and to link it to improved cognitive performance.

Based on a highly selected population of healthy university students, this randomised, double-blind, controlled, intervention study presented some limitations that may induce potential bias in the analyse of product effect. Indeed, polyphenol intake from food, beverages, or supplements may affect the efficacy endpoints as well as circulating metabolites of the study product if participants do not follow instructions regarding food intake. However, compliance with these instructions and food diary have been checked by the research team and no major deviation was reported.

## 5. Conclusions

In healthy young adults, acute supplementation with a PEGB was efficient at improving cognitive performance and in particular working memory and attention during a highly effortful cognitive challenge. Although further work is necessary to understand the underlying mechanisms of action of these compounds, findings emanating from this study provide further substantiation for the development of EFSA/FDA health claims on specific polyphenol containing products and cognitive enhancement [55]. Consumption of PEGB coupled with a healthy lifestyle may be a safe alternative to acutely improve cognitive performance during a sustained cognitive effort.

## Figures and Tables

**Table 1:** Composition of the polyphenol-rich extract derived from grape and blueberry tested on healthy young adults.

Compounds (%)	Mean	SD
Total flavonoids (flavan-3-ols, flavonols and anthocyanins)	43.4	2.1
Flavan-3-ols monomers	20.6	1.3
Oligomers (DP < 5)	22.5	4.5
Flavonols (quercetin, glycosylated derivatives)	0.2	0.1
Anthocyanins	0.1	0
Phenolic acids (chlorogenic acids, gallic acids)	0.5	0.1
Stilbenes (trans-resveratrol)	0.1	0.1

SD: standard deviation; DP: degree of polymerisation.

**Table 2:** Demographic and medical characteristics of the subjects at inclusion (*n* = 30).

Characteristics	Mean	SD
Age (years)	22	1.7
Gender (ratio male/female)	0.9	
Body mass index (kg/m²)	21.3	2.2
Systolic blood pressure (mmHg)	115.3	9.6
Diastolic blood pressure (mmHg)	74.7	7.6
Heart rate (bpm)	77.6	14.8
Normal sleeping duration (h)	7.9	0.8

SD: standard deviation.

**Table 3:** Mean ± SE scores for STS, SSS, and RVIP at the beginning of cognitive challenge and change from first score during the cognitive challenge and according to the treatment group.

Measures	Treatment	*n*	Scores 90 min Post-Product Ingestion		Variation of CDB Scores										*p*-Values	
					22 min		33 min		44 min		55 min		66 min			
			Mean	SE	Mean	SE	Mean	SE	Mean	SE	Mean	SE	Mean	SE	Treatment Effect	Repetition Effect
STS (total answers)	Placebo	30	62.8	3.7	3.5	1.5	3.3	1.7	2.3	2.2	5.4	1.9	6.1	2.3	**0.001**	**0.01**
	PEGB	30	59.3	3.9	5.1	2.0	6.4	2.2	8.5	2.2	7.7	1.8	11.8	2.2		
STS (nb correct)	Placebo	30	61.3	3.7	3.7	1.5	2.9	1.7	1.6	2.3	5.3	2	5.2	2.6	**<0.001**	0.101
	PEGB	30	57.3	3.9	5.6	2.1	6.7	2.3	8.9	2.3	7.7	1.9	11.7	2.2		
STS (nb error)	Placebo	30	1.4	0.3	−0.2	0.3	0.4	0.3	0.7	0.4	0.1	0.4	0.8	0.5	**0.041**	0.123
	PEGB	30	1.0	0.4	−0.5	0.4	−0.3	0.4	−0.4	0.4	0.0	0.5	0.1	0.4		
STS% correct	Placebo	30	97.5	2.6	0.5	0.5	−0.5	0.5	−1.1	0.8	0.4	0.6	−1	0.9	**0.001**	0.126
	PEGB	30	96.1	3.7	1.7	0.7	1.2	0.7	1.5	0.6	0.8	0.8	0.8	0.7		
SSS (total answers)	Placebo	30	32.8	2.5	1.2	0.8	1.5	0.8	1.5	0.8	2.5	0.8	3.8	1.0	0.793	**<0.001**
	PEGB	30	32.3	2.2	0.6	1.0	1.3	0.9	1.5	1.0	3.5	1.1	4.3	1.0		
SSS (nb correct)	Placebo	30	31.5	2.4	1.1	0.8	1.3	0.9	0.6	0.9	2.5	0.9	3.8	1.0	0.995	**<0.001**
	PEGB	30	31.2	2.2	0.4	1.0	1.0	1.0	1.4	1.3	2.8	1.2	3.8	1.0		
SSS (nb error)	Placebo	30	1.3	0.3	0.1	0.4	0.2	0.3	0.8	0.4	0.1	0.3	−0.1	0.3	0.536	0.643
	PEGB	30	1.2	0.2	0.2	0.3	0.3	0.4	0.1	0.4	0.7	0.4	0.5	0.2		
SSS% correct	Placebo	30	95.9	7.1	−1.5	1.3	−1.4	1.3	−3.8	1.6	0.1	1.0	0.5	1.1	0.684	0.367
	PEGB	30	95.8	5.6	−0.2	1.4	−0.9	1.4	−0.8	1.6	−1.6	1.2	−0.9	0.6		
RVIP FA	Placebo	30	2.1	0.4	−0.1	0.3	1.2	0.4	1.0	0.6	0.7	0.4	1.0	0.5	0.300	0.098
	PEGB	30	2.0	0.3	0.3	0.3	0.5	0.3	0.6	0.4	0.3	0.3	0.6	0.4		
RVIP RT (s)	Placebo	30	492.4	8.4	−0.5	5.8	−0.6	6.4	10.2	6.7	−1.1	5.6	3.7	7.3	0.846	0.411
	PEGB	30	490.0	9.2	3.6	3.5	4.6	5.8	−1.2	7.6	−2.9	6.2	3.8	7.9		

A total of six CDB measures (every 11 min) were performed during the cognitive challenge. *p*-values associated with treatment and repetition effect are presented. *p*-values in bold are statistically significant. STS: serial three subtraction, SSS: serial seven subtraction, RVIP: rapid visual information processing, FA: false alarm, (s): second, nb: number, SE: Standard error. *n* is the number of participants included in the final analysis. Statistically significant *p*-values are in bold.

## Supplementary Materials

The following are available online at https://www.mdpi.com/2076-3921/8/12/650/s1, Figure S1: Mean ± SE performance variation scores according to treatment group along CDB repetitions for (**a**) STS, (**b**) RVIP and (**c**) SSS.
