# Supplementary material for: Acute Intake of a Grape and Blueberry Polyphenol-Rich Extract Ameliorates Cognitive Performance in Healthy Young Adults During a Sustained Cognitive Effort
Source: Antioxidants (Basel). 2019 Dec 17;8(12):650. doi: 10.3390/antiox8120650 (PMC6943592; doi:10.3390/antiox8120650)
Supplement: Supplementary file 1 [file antioxidants-08-00650-s001.pdf]

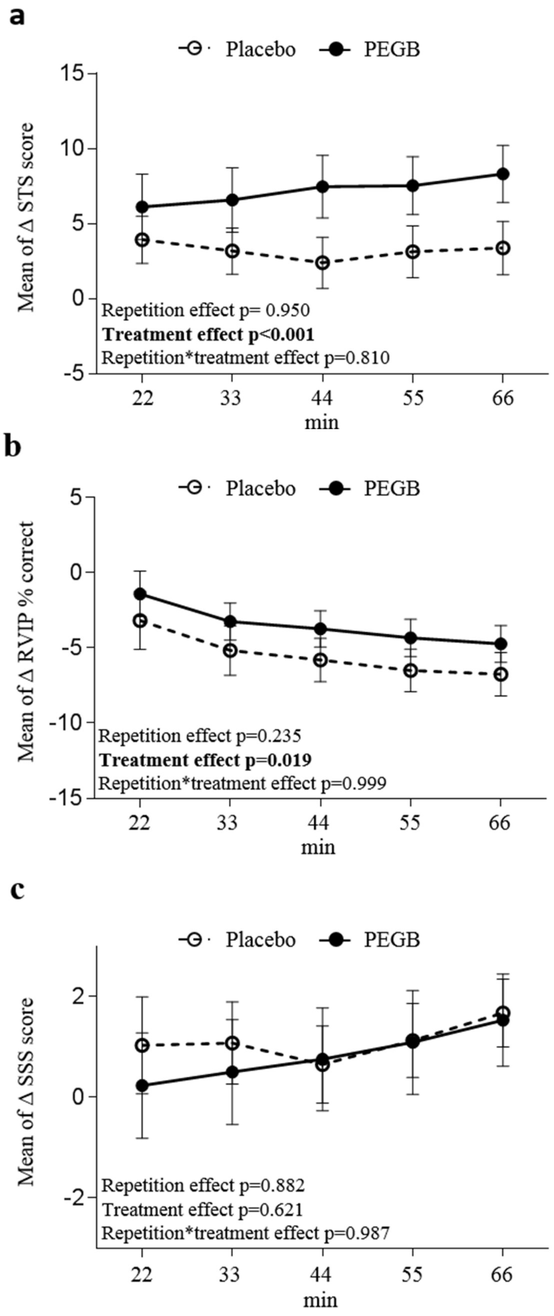

Figure S1: Mean ± SE performance variation scores according to treatment group along CDB repetitions for (**a**) STS, (**b**) RVIP and (**c**) SSS. A total of 6 CDB measures (every 11 min) were performed during the cognitive challenge. Raw data score obtained from STS, RVIP, SSS along the different CDB repetitions were firstly expressed as change from first repetition (Δ). Then an incremental mean approach was used. For each CDB repetition (22 min, 33min, 44min, 55 min and 66 min from start of cognitive effort), changes from first score were averaged with previous repetitions to obtain mean of variation scores. *p*-values generated by the linear-mixed models are reported for the effects of treatment, repetition and treatment x repetition. P values in bold are statistically significant. STS: Serial Three Subtraction, SSS: Serial Seven Subtraction, RVIP: Rapid visual information processing, SE: Standard error.
